# FITM2‐Related Siddiqi Syndrome in Two Iranian Siblings

**DOI:** 10.1002/ccr3.71303

**Published:** 2025-10-15

**Authors:** Raha Ahmadi, Mohammad Javad Bavarsad, Meysam Feizollah Jani, Reza Azizimalamiri

**Affiliations:** ^1^ Department of Pediatric Neurology, Golestan Medical, Educational, and Research Center Ahvaz Jundishapur University of Medical Sciences Ahvaz Iran

**Keywords:** deafness, dystonia, FITM2, ichthyosis, Iran, Siddiqi syndrome

## Abstract

We report the first two Iranian siblings with Siddiqi syndrome, carrying a novel likely pathogenic FITM2 variant. Both presented with hallmark features, including early‐onset sensorineural hearing loss, severe generalized dystonia, growth failure, and ichthyosis of the lower limbs, expanding the geographic and genetic spectrum of this rare disorder.

## 1 Introduction

Siddiqi syndrome is an ultra‐rare, autosomal recessive neurodevelopmental disorder characterized by a progressive and complex clinical phenotype. Its core features include early‐onset sensorineural hearing loss, generalized dystonia, motor regression, growth failure, and ichthyosis, often accompanied by additional manifestations such as intellectual disability and peripheral neuropathy. The disorder is caused by biallelic pathogenic variants in the Fat Storage‐Inducing Transmembrane Protein 2 (FITM2) gene, which plays a critical role in lipid metabolism and energy homeostasis [1].

FITM2 encodes a transmembrane protein essential for the formation and maintenance of intracellular lipid droplets—organelles vital for lipid storage and trafficking. Disruption of this pathway impairs lipid metabolism, particularly in neurons, which are highly dependent on lipid signaling and myelination. Consequently, FITM2 dysfunction is thought to lead to neurodegeneration through abnormal cellular signaling, disrupted myelin maintenance, and altered synaptic transmission. Clinically, patients with Siddiqi syndrome typically present in early infancy with progressive hearing impairment, hypotonia, and dystonia, which may evolve into severe motor disability. Growth parameters—including weight, height, and head circumference—are frequently below the 3rd percentile, reflecting the syndrome's systemic involvement. Ichthyosis, usually affecting the lower limbs, is another consistent feature, although its severity may vary. Despite the presence of a recognizable clinical triad—deafness, dystonia, and ichthyosis—the syndrome remains underdiagnosed due to its rarity and clinical overlap with other neurogenetic conditions [1, 2, 3, 4].

Although the precise pathomechanisms remain incompletely understood, emerging evidence suggests that FITM2‐related neurodegeneration results from disrupted neuronal lipid homeostasis. While histopathologic data are limited, neuroimaging often reveals structural abnormalities—particularly involving the basal ganglia—that support the movement disorder phenotype and implicate FITM2 in essential developmental processes of the central nervous system [1, 2, 4].

To date, only 10 patients from five families have been reported worldwide, originating from Pakistan (five siblings), the United States (one case), Germany (one), China (one), and Russia (two siblings). Most individuals were born to consanguineous parents, consistent with autosomal recessive inheritance and suggesting a role for founder mutations or regional genetic drift. The extreme rarity of confirmed cases, combined with the severity of the clinical presentation, highlights the need for greater awareness—particularly in regions with high rates of consanguinity. Here, we report the first two Iranian siblings diagnosed with Siddiqi syndrome. Both were born to consanguineous parents from southwestern Iran and carry a novel, likely pathogenic homozygous variant in FITM2. This report expands both the genetic and geographic spectrum of FITM2‐related neurodevelopmental disorders [1, 2, 3, 4, 5, 6].

## 2 Clinical Report

### 2.1 First Sibling

The first sibling, a 12‐year‐old girl, was born preterm following a complicated out‐of‐hospital delivery characterized by meconium‐stained amniotic fluid and low APGAR scores. She required immediate resuscitation and was admitted to the neonatal intensive care unit (NICU) for 10 days, during which a single seizure episode was observed. Brain imaging performed during the NICU stay was unremarkable. By 9 months of age, developmental concerns emerged, including persistent axial hypotonia, inability to achieve head control, brisk deep tendon reflexes, and dystonic posturing. Auditory brainstem response (ABR) testing confirmed severe bilateral sensorineural hearing loss (~100 dB). Over the following years, her motor function progressively declined. By age 12, she was non‐verbal, non‐ambulatory, and fully dependent for all activities of daily living. She exhibited profound generalized dystonia with opisthotonic posturing, and her weight, height, and head circumference remained below the 3rd percentile. Dermatologic examination revealed ichthyosis localized to the lower limbs.

### 2.2 Neurological Examination

Initial neurological evaluation at 9 months revealed an alert but poorly interactive infant with no visual tracking or response to sound. Cranial nerve examination showed reactive pupils and a normal fundus, with no gaze palsy, ptosis, or facial asymmetry. Axial hypotonia was prominent, while appendicular tone was mildly increased. Deep tendon reflexes were brisk and symmetric in all extremities, and bilateral plantar responses were extensor. An asymmetric tonic neck reflex was noted. Sensory testing was limited but showed intact withdrawal to noxious stimuli. Coordination and gait could not be assessed. Hearing impairment was later confirmed by ABR.

**FIGURE 1:**
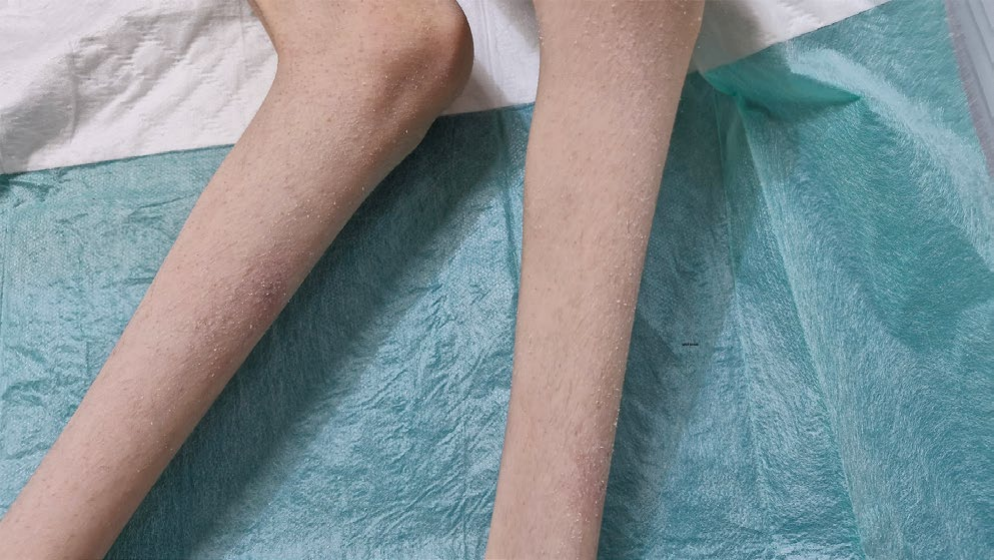
Image showing the shins of the female patient demonstrating bilateral ichthyosis.

**FIGURE 2:**
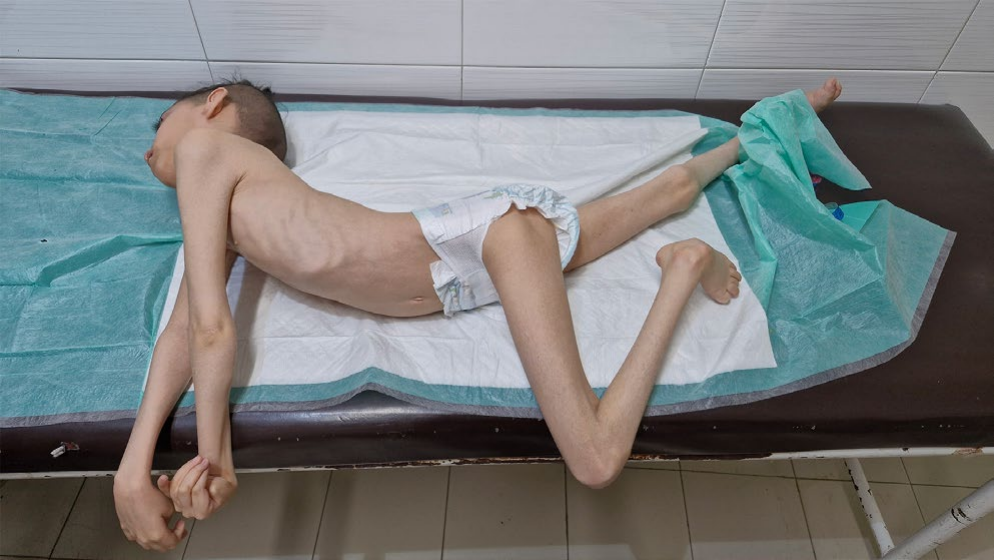
Image of the female patient showing severe generalized dystonia.

Over the next several years, she experienced progressive neurological deterioration. By age 5, she had developed sustained opisthotonic posturing with contractures at the knees and elbows. She remained non‐verbal, unable to sit, roll, or reach. Reflexes remained brisk with bilateral ankle clonus. Dermatologic evaluation at this stage confirmed ichthyosis affecting the shins (see Figure 1). By age 12, she demonstrated severe truncal hyperextension, oromotor dysfunction, drooling, and continued opisthotonos (see Figure 2). Cranial nerve function could not be fully assessed but remained grossly intact. No cerebellar signs, seizures, or spasticity were observed. The clinical course suggested a progressive, non‐spastic dystonic encephalopathy with dermatologic and auditory involvement.

### 2.3 Differential Diagnosis

An extensive diagnostic workup was undertaken. Dyskinetic cerebral palsy was initially considered due to the perinatal complications; however, the progressive nature of symptoms, normal MRI, absence of spasticity, and similar presentation in her sibling with no perinatal insult rendered this diagnosis unlikely. Metabolic and mitochondrial disorders (e.g., glutaric aciduria type I, biotinidase deficiency) were ruled out through normal biochemical workup and unremarkable neuroimaging. Leukodystrophies such as Pelizaeus‐Merzbacher disease and metachromatic leukodystrophy were excluded due to the absence of white matter changes. Neurodegeneration with brain iron accumulation (NBIA) was considered due to the dystonia, but no basal ganglia iron deposition was seen on MRI. Syndromic ichthyoses such as Refsum disease and Sjögren‐Larsson syndrome were ruled out by the absence of retinitis pigmentosa, spasticity, and leukoencephalopathy. Ultimately, the combination of early‐onset deafness, progressive dystonia, ichthyosis, and consanguinity pointed to a genetic neurodevelopmental syndrome—later confirmed as Siddiqi syndrome following genetic analysis of her younger brother.

### 2.4 Second Sibling

The second sibling, an 8‐year‐old boy, was born via cesarean section due to oligohydramnios, with no perinatal complications. Early developmental delay became evident by 6 months, including poor head control and hypotonia. By 12 months, he exhibited generalized dystonia and poor auditory responsiveness. Neurological evaluation revealed axial hypotonia with distal hypertonia, dystonic posturing, and brisk reflexes. Cranial nerves were intact. Brain MRI was normal, showing no structural or white matter abnormalities. ABR testing confirmed severe bilateral sensorineural hearing loss. Over time, he failed to achieve independent sitting or speech and developed progressive dystonia and feeding difficulties. At age 8, all growth parameters remained below the 3rd percentile. He also showed ichthyosis over the anterior shins and dorsum of the feet.

### 2.5 Neurological Examination

At 6 months, he was alert but had poor social interaction and no auditory responses. Pupils were reactive, and cranial nerves were intact. He demonstrated generalized hypotonia with poor antigravity movement and truncal weakness. DTRs were brisk and symmetric; plantar responses were bilaterally extensor. No dyskinesia or ataxia was noted. Although sensory testing was limited, withdrawal to pain was preserved. Mild dryness was observed on the lower limbs. ABR confirmed profound bilateral hearing loss.

By age 3, his dystonia had progressed, particularly in the trunk and proximal limbs. He remained unable to sit, reach, or follow commands. Visual tracking was inconsistent. By age 8, dystonia had become severe and persistent, with prominent truncal rigidity and opisthotonic posturing. He remained non‐verbal and completely dependent. Oromotor dysfunction led to drooling and feeding issues. Cranial nerve exam was unremarkable. Reflexes remained brisk with sustained clonus and bilateral Babinski signs. No cerebellar signs or clinical seizures were observed. Skin findings were consistent with non‐bullous ichthyosis.

### 2.6 Differential Diagnosis

As in his sister, cerebral palsy was considered but ruled out due to a lack of perinatal insult and a progressive trajectory. Leukodystrophies, mitochondrial disorders, and NBIA were excluded based on imaging, biochemical screening, and clinical presentation. Syndromic ichthyoses and auditory neuropathies were considered but could not account for the full constellation of features. The combination of sensorineural deafness, progressive dystonia, global developmental delay, ichthyosis, and normal imaging in the setting of consanguinity and recurrence in a sibling strongly suggested an autosomal recessive neurogenetic condition such as Siddiqi syndrome.

### 2.7 Genetic Analysis

Given the highly similar phenotypes in both siblings, whole exome sequencing (WES) was performed in the younger sibling as a first‐tier diagnostic tool. A novel homozygous frameshift variant was identified in the FITM2 gene: c.114dupC (p.Lys39Glnfs*113), predicted to result in a premature stop codon and truncated protein product, leading to loss‐of‐function. Loss‐of‐function variants are a known pathogenic mechanism in Siddiqi syndrome.

According to the ACMG 2015 guidelines, this variant meets the following criteria: PVS1: Null (frameshift) variant in a gene where loss of function is a known mechanism of disease. PM2: Absent from population databases including gnomAD and ExAC. PP4: Phenotype highly specific for FITM2‐related Siddiqi syndrome (deafness, dystonia, ichthyosis).

Thus, the variant was classified as likely pathogenic. Sanger sequencing confirmed homozygosity in both affected siblings and heterozygosity in both parents, consistent with autosomal recessive inheritance. Management for both siblings remained supportive, focusing on nutritional rehabilitation, physiotherapy, dermatologic care, and audiologic evaluation. Genetic counseling was provided to the family [7].

## 3 Discussion

Siddiqi syndrome should be considered in the differential diagnosis of children presenting with global developmental delay, early‐onset generalized dystonia, sensorineural hearing loss, and ichthyosis—particularly in consanguineous populations, once other etiologies such as metabolic disorders, cerebral palsy, and structural brain abnormalities have been excluded. First described by Zazo Seco et al. in a consanguineous Pakistani family with five affected siblings, Siddiqi syndrome is a rare autosomal recessive neurodevelopmental disorder caused by biallelic pathogenic variants in the FITM2 gene (c.4G>T, p.Glu2*). Since that initial report, only a limited number of cases have been documented worldwide, emphasizing both the extreme rarity and the phenotypic consistency of the condition [1].

FITM2 encodes the fat storage‐inducing transmembrane protein 2, which plays a critical role in lipid droplet formation and intracellular lipid homeostasis. Loss‐of‐function mutations in FITM2 have been linked to impaired neuronal lipid metabolism, which may disrupt cellular signaling, myelin maintenance, and synaptic transmission—processes essential for neurodevelopment and function. Affected individuals typically present with a stereotyped phenotype that includes early‐onset sensorineural deafness, severe generalized dystonia, motor regression or delay, ichthyosis (often localized to the lower extremities), and significant growth failure, including microcephaly, short stature, and underweight. These features were consistently observed in our patients and align closely with previously reported cases [1, 2, 3, 4].

The clinical presentation in our siblings prompted a broad differential diagnosis. Dyskinetic cerebral palsy was initially suspected in the older sibling due to her complicated perinatal course. However, the progressive nature of symptoms, normal brain MRI, and similar phenotype in the younger sibling—who had an uneventful perinatal history—strongly argued against a static encephalopathy. Leukodystrophies, such as metachromatic leukodystrophy and Pelizaeus‐Merzbacher disease, were considered due to hypotonia and motor regression, but the absence of white matter abnormalities, cognitive decline, and spasticity made these diagnoses unlikely. Mitochondrial and metabolic disorders, including glutaric aciduria type I, biotinidase deficiency, and mitochondrial encephalopathies, were excluded based on normal metabolic profiles, the lack of systemic involvement, and stable neuroimaging findings.

NBIA was considered given the presence of early‐onset dystonia and progressive motor dysfunction, but MRI showed no evidence of iron deposition in the basal ganglia. Syndromes involving ichthyosis and neurologic impairment, such as Refsum disease, Sjögren‐Larsson syndrome, and Chanarin‐Dorfman syndrome, were also explored. However, the absence of associated findings—such as retinitis pigmentosa, spasticity, hepatic involvement, and specific biochemical markers—made these conditions unlikely. Ultimately, the diagnosis was guided by the triad of early‐onset sensorineural hearing loss, progressive generalized dystonia, and non‐bullous ichthyosis, in the context of consanguinity, negative metabolic and neuroimaging workup, and a shared presentation in both siblings. The identification of a novel, homozygous, likely pathogenic frameshift variant in FITM2 confirmed the diagnosis of Siddiqi syndrome, consistent with previous reports.

To date, only 10 patients from five families have been described in the literature—originating from Pakistan (five siblings), the United States (one case), Germany (one), China (one), and Russia (two siblings). The original Pakistani family carried a homozygous nonsense variant (c.4G>T, p.Glu2*). The American case involved compound heterozygous loss‐of‐function variants (c.39dupC and c.652C>T). A German patient had a homozygous missense variant (c.694G>A, p.Gly232Arg), while the Chinese patient harbored a homozygous frameshift variant (c.611_612dupTG, p.Met205fs). Most recently, two Russian siblings were found to have a homozygous missense variant (c.452A>G, p.Asp151Gly) [1, 2, 3, 4, 5].

These geographically dispersed but clinically similar cases suggest that FITM2‐related Siddiqi syndrome is likely underdiagnosed due to its rarity and phenotypic overlap with other neurodevelopmental and neurodegenerative disorders. Our report contributes to the expanding clinical and genetic spectrum of this disorder through the identification of a novel frameshift variant—c.114dupC (p.Lys39Glnfs*113)—in two affected siblings of Iranian origin.

Genotype–phenotype correlations from the literature suggest that truncating variants (e.g., c.39dupC in the U.S. case and c.611_612dupTG in the Chinese case) are associated with more severe phenotypes, including early‐onset dystonia, profound developmental delay, and growth failure. In contrast, patients harboring missense variants (e.g., p.Asp151Gly and p.Gly232Arg) may exhibit relatively milder phenotypes, although data remain limited. Our two siblings, both homozygous for a novel truncating variant, exhibited a severe clinical course characterized by early‐onset sensorineural deafness, generalized dystonia, ichthyosis, and significant growth impairment—further supporting a potential genotype–phenotype correlation.

A summary of the reported cases, including our own, is provided in Table 1, which outlines clinical features, variant type, inheritance patterns, neuroimaging findings, and phenotypic severity across all known patients.

**TABLE 1:** Summary of reported cases of FITM2‐related Siddiqi syndrome.

Case no.	Country	Genetic variant(s)	Variant type	Inheritance	Neuroimaging	Deafness	Dystonia	Ichthyosis	Growth failure	References
1–5	Pakistan	c.4G>T (p.Glu2*)	Nonsense	Homozygous	MRI was done in two siblings and both were normal	Yes	Yes	Yes	Yes	Zazo Seco et al. [1]
6	USA	c.39dupC/c.652C>T	Frameshift/Nonsense	Compound Heterozygous	Hypoplasia of inferior vermis	Yes	Yes	Yes	Yes	Shakir et al. [2]
7	Germany	c.694G>A (p.Gly232Arg)	Missense	Homozygous	Not reported	Yes	Yes	Mild	Yes	Riedhammer et al. [3]
8	China	c.611_612dupTG (p.Met205fs)	Frameshift	Homozygous	Bilateral globus pallidus T2 hyperintensity	Yes	Yes	Yes	Yes	Lin et al. [4]
9–10	Russia	c.452A>G (p.Asp151Gly)	Missense	Homozygous	Not reported	Yes	Yes	Mild	Mild	Rudenskaya et al. [5]
11–12	**Iran (Our Cases)**	c.114dupC (p.Lys39Glnfs*113)	Frameshift	Homozygous	Normal in both siblings	Yes	Yes	Yes	Yes	**Current study**

This report highlights several key clinical implications. First, Siddiqi syndrome should be recognized as a distinct clinical entity in children presenting with the characteristic triad of deafness, dystonia, and ichthyosis, particularly in consanguineous families. Second, early genetic diagnosis is critical for enabling appropriate supportive care, family counseling, and informed reproductive decisions. Although no disease‐modifying treatments currently exist, early interventions—such as cochlear implantation, pharmacologic and rehabilitative support for dystonia, and nutritional management—may improve functional outcomes and quality of life.

Finally, our findings underscore the importance of expanding awareness of FITM2‐related disorders and support the need for future studies to clarify their molecular pathophysiology. In particular, functional analyses comparing truncating versus missense variants may yield valuable insights into disease mechanisms and progression. Future collaborative efforts, including multicenter registries and longitudinal studies, will be essential to delineate the full clinical spectrum, establish natural history data, and ultimately explore therapeutic targets for this debilitating condition.

## Author Contributions

**Mohammad Javad Bavarsad:** data curation, investigation, writing – original draft. **Raha Ahmadi:** data curation, investigation, writing – original draft. **Meysam Feizollah Jani:** investigation, supervision, writing – review and editing. **Reza Azizimalamiri:** conceptualization, methodology, project administration, supervision, writing – review and editing.

## Ethics Statement

This study was conducted in accordance with the principles outlined in the Declaration of Helsinki. Ethical approval was obtained from the Ethics Committee of Ahvaz Jundishapur University of Medical Sciences.

## Consent

Written informed consent was obtained from the parents of both affected individuals for participation in the study and for publication of anonymized clinical data and images.

## Conflicts of Interest

The authors declare no conflicts of interest.

## Data Availability

The data supporting the findings of this study are available from the corresponding author upon reasonable request. Due to privacy and ethical restrictions, individual‐level genetic data and patient‐identifiable clinical data are not publicly available.

## References

[1] C. Zazo Seco , A. Castells‐Nobau , S. H. Joo , et al., “A Homozygous FITM2 Mutation Causes a Deafness‐Dystonia Syndrome With Motor Regression and Signs of Ichthyosis and Sensory Neuropathy,” Disease Models and Mechanisms 10, no. 2 (2017): 105–118, 10.1242/dmm.026476. 28067622 PMC5312003

[2] A. Shakir , A. F. Wadley , G. Purcarin , and K. J. Wierenga , “The First Case of Deafness‐Dystonia Syndrome due to Compound Heterozygous Variants in FITM2,” Clinical Case Reports 6, no. 9 (2018): 1815–1817, 10.1002/ccr3.1719. 30214770 PMC6132111

[3] K. M. Riedhammer , G. S. Leszinski , S. Andres , G. Strobl‐Wildemann , and M. Wagner , “First Replication That Biallelic Variants in FITM2 Cause a Complex Deafness‐Dystonia Syndrome,” Movement Disorders 33, no. 10 (2018): 1665–1666, 10.1002/mds.27481. 30288795

[4] Y. Lin , W. Zhang , D. Li , et al., “The First Chinese Case of Siddiqi Syndrome Caused by a Homozygous FITM2 Variant,” Clinical Genetics 102, no. 3 (2022): 246–247, 10.1111/cge.14178. 35754111

[5] G. E. Rudenskaya , F. M. Bostanova , A. S. Medvedeva , E. E. Lotnik , P. A. Chausova , and O. A. Shchagina , “A Case of Rare Hereditary Siddiqi Syndrome With Novel Neuropsychiatric Signs,” Zhurnal Nevrologii I Psikhiatrii Imeni S. S. Korsakova 124, no. 12 (2024): 171–176, 10.17116/jnevro2024124121171. 39731388

[6] A. U. Neshan , S. M. U. Tabatabaei , M. U. Mohammadi , M. U. Narooie‐Nejad , and F. U. Behmanesh Pour , “The Prevalence and Patterns of Consanguineous Marriages and Associated Factors in Southeast of Iran,” Gene, Cell and Tissue 11 (2024): e147204, 10.5812/gct-147204.

[7] S. Richards , N. Aziz , S. Bale , et al., “Standards and Guidelines for the Interpretation of Sequence Variants: A Joint Consensus Recommendation of the American College of Medical Genetics and Genomics and the Association for Molecular Pathology,” Genetics in Medicine 17, no. 5 (2015): 405–424. 25741868 10.1038/gim.2015.30 PMC4544753

