# Correction to “FITM2‐Related Siddiqi Syndrome in Two Iranian Siblings”

**DOI:** 10.1002/ccr3.72062

**Published:** 2026-02-12

Ahmadi R, Bavarsad MJ, Feizollah Jani M, Azizimalamiri R. *Clinical Case Reports*. 2025;13(10):e71303.

In Table 1, the Russian cases were incorrectly marked as having dystonia (“Yes”). This was an error. The Russian cases did not have dystonia, and the correct entry should be “No.”

We apologize for this error.

